# Urinary Cytokines in Predicting Intradetrusor Onabotulinumtoxin‐A Response

**DOI:** 10.1002/nau.70261

**Published:** 2026-03-15

**Authors:** Marina Guirguis Hanna, Ashti M. Shah, Wuqi Li, Ervin Sejdic, Megan Bradley, Stephanie W. Zuo, Amanda M. Artsen

**Affiliations:** ^1^ Department of Obstetrics & Gynecology, Division of Urogynecology Inova Health System Falls Church Virginia USA; ^2^ Department of Obstetrics, Gynecology & Reproductive Services Division of Urogynecology, UPMC Pittsburgh Pennsylvania USA; ^3^ University of Pittsburgh School of Medicine Pittsburgh Pennsylvania USA; ^4^ Edward S. Rogers Sr. Department of Electrical & Computer Engineering University of Toronto Ontario Canada; ^5^ North York General Hospital Ontario Canada; ^6^ Magee‐Womens Research Institute Pittsburgh Pennsylvania USA; ^7^ Department of Obstetrics and Gynecology, Division of Urogynecology University of Virginia Charlottesville Virginia USA

**Keywords:** asymptomatic bacteriuria, biomarkers, cytokines, inflammation, Onabotulinumtoxin‐A

## Abstract

**Purpose:**

The impact of asymptomatic bacteriuria and inflammation on response to Onabotulinumtoxin‐A for overactive bladder is poorly understood. This work compares baseline differences in asymptomatic bacteriuria status and urinary inflammatory markers between women who respond to Onabotulinumtoxin‐A and those who do not.

**Materials and Methods:**

Women undergoing intradetrusor Onabotulinumtoxin‐A injection for refractory overactive bladder submitted a catheterized urine sample to assess for asymptomatic bacteriuria and quantification of 12 urinary cytokines and chemokines at the time of injection. Clinical responders were defined as meeting a minimal clinical difference of > 11 on the Urinary Distress Inventory Short Form after treatment. The proportion of asymptomatic bacteriuria and concentration of urinary biomarkers in Onabotulinumtoxin‐A responders compared to non‐responders were analyzed.

**Results:**

Of the 75 participants, 45 (60%) were Onabotulinumtoxin‐A responders. Responders did not differ in baseline characteristics apart from age (66.0 ± 14.2 [responders] vs. 74.3 ± 7.8 years [non‐responders], *p* < 0.01). There was no difference in asymptomatic bacteriuria rates in responders (37.8%) versus non‐responders (40.7%, *p* = 0.09). No single inflammatory marker was predictive of treatment response when controlling for age. Decision tree analysis allowed for 80% classification accuracy of responders. MCP1 and IP10 were major features in the decision tree analysis, but their impact varied by age. Adding asymptomatic bacteriuria to the decision tree analysis did not improve classification nor function as an important predictive feature.

**Conclusions:**

Asymptomatic bacteriuria does not affect Onabotulinumtoxin‐A response. Older women were less likely to respond to Onabotulinumtoxin‐A treatment and experienced a differential impact of inflammatory cytokines.

## 1 Introduction

Overactive bladder (OAB) is a debilitating syndrome of urinary urgency with or without urgency urinary incontinence (UUI) that affects 17% of women over the age of 45 and up to 40% of women over the age of 75 in the United States [1]. When monotherapy or non‐invasive therapies are not effective for the management of OAB, minimally invasive procedures such as posterior tibial nerve stimulation, sacral neuromodulation, and intradetrusor Onabotulinumtoxin‐A (BTX‐A) injections are utilized [2]. Research has demonstrated that although intradetrusor BTX‐A injections can significantly improve symptoms of refractory OAB and UUI in many patients, up to 39% of women experience < 50% improvement [3].

It is posited that inadequate symptom improvement with BTX‐A is due to a combination of bladder dynamics, such as high maximum detrusor pressure and low compliance; severity of symptoms; and systemic wellbeing [4]. Widespread and local inflammation, as seen with aging, increased comorbidities, and higher body mass index (BMI) [5], may impact treatment response through changes in urothelial signaling or urothelial damage [6].

Furthermore, an inflamed bladder microenvironment can have a profound effect on the bladder microbiome and bacterial homeostasis within the bladder [7]. Inflammation can result in alterations in the host response to bacteria, increasing the risk of asymptomatic bacteriuria (ASB), particularly in older women [8]. At present, it is unclear how the characteristics of the inflammatory environment and presence of ASB affect OAB treatment response with BTX‐A.

A prognostic biomarker for treatment response would not only allow for better individualized treatment plans but also improve our understanding of the underlying mechanisms of disease. Currently, there is significant heterogeneity among trials investigating biomarker associations with OAB [9], and very few studies utilize biomarkers to predict response to treatment [10, 11].

The primary objective in this study is to characterize the response to BTX‐A based on ASB status and baseline differences in bladder inflammation. This information will allow clinicians to understand the potential impact of ASB in patients needing BTX‐A. We hypothesized that patients with poor response to intradetrusor BTX‐A are more likely to have bacteriuria and higher levels of urinary pro‐inflammatory markers compared to those with an effective treatment response. Secondarily, we aimed to examine characteristics and evidence of inflammation in those with and without ASB to better understand this population. We hypothesized that ASB would be associated with increased markers of inflammation.

## 2 Materials and Methods

### 2.1 Patient Recruitment

This is a prospective cohort study of patients who were assigned female at birth undergoing intradetrusor BTX‐A injection for refractory OAB from 2021 to 2023. This study was approved by the University of Pittsburgh Institutional Review Board (#21010132). Patients were included if they were at least 18 years of age, failed first‐ and second‐line therapy for refractory OAB, and were planned to undergo an injection of 100−300 units of intravesical BTX‐A. Exclusion criteria included neurogenic bladder, pre‐procedure urinary retention defined as a postvoid residual of > 300 mL, and inability to comply with study procedures. Prior history of BTX‐A injection for refractory OAB was allowed. Patients were also excluded if they answered “Yes” to the question “Do you feel like you have a urinary tract infection today?”

### 2.2 Collected Measures

Demographic characteristics and medical history were collected for all participants. Participants completed the Urinary Distress Inventory Short Form (UDI‐6) on the day of injection and again 30 days after BTX‐A injection. The decision for reassessment 30 days after injection was made as the typical onset of action for BTX‐A is 10−14 days, and by assessing treatment response at 30 days, we could ensure that all participants had adequate time for the BTX‐A to take effect [12].

### 2.3 Urine Collection and Processing

Immediately prior to BTX‐A injection, transurethral catheterized specimens were collected for urine culture and inflammatory cytokine analysis. An aliquot of urine was sent for standard urine culture, with the remainder placed on ice and taken to the laboratory within 4 h of collection. An aliquot was sent for standard urine culture with bacteriuria defined as > 10 000 colony‐forming units of 1−2 organisms. After decanting, the urine was centrifuged at 1000*g* for 10 min. The supernatant from the urine sample was then then aliquoted and stored at −80°C until cytokine quantification.

### 2.4 Electrochemiluminescence Multiplex

The following analytes were quantified from urine samples using a hypothesis‐driven set of biomarkers identified by validated electrochemiluminescence multiplex assays (Mesoscale Discovery, Rockville, MD V‐Plex Pro‐inflammatory, Chemokine, and Angiogenesis Panels: basic fibroblastic growth factor (bFGF), vascular endothelial growth factor (VEGF), interferon gamma (IFNγ), interleukin‐10 (IL‐10), interleukin‐12 protein of 70 kD (IL‐12p70), interleukin‐13 (IL‐13), interleukin‐4 (IL‐4), interleukin‐6 (IL‐6), interleukin‐8 (IL‐8), tumor necrosis factor alpha (TNF‐α), IFNγ induced protein‐10 (IP‐10), and monocyte chemoattractant protein‐1 (MCP‐1). Growth‐regulated protein alpha (GRO‐α), beta‐Nerve Growth Factor (β‐NGF), and Brain‐Derived Neurotrophic Factor (BDNF) were assessed using a custom multiplex electrochemiluminescence panel (U‐Plex, Mesoscale Discovery, Rockville, MD). These markers were chosen to encompass an array of pro‐ and anti‐inflammatory assays and include promising biomarkers previously identified in the literature [9, 10, 11, 13, 14, 15, 16, 17, 18]. Assays were performed in duplicate and normalized to urinary creatinine in mg/dL. Validated panels have coefficients of variation (CVs) < 20% and antibodies with < 1% non‐specific binding. If less than 10% of samples were below the observed lower limit of detection, the value was considered to be the lower limit of detection/2. Technical replicate variation was assessed, and values with CVs > 20% were excluded (< 3%). Quantification limits are provided in Supporting Information S1: Table 1.

### 2.5 Statistical Analysis

The primary outcome was the proportion of ASB in responders compared to non‐responders. We defined responder status as a change of ≥ 11 points on the UDI‐6 questionnaire, which has been reported as the minimally important clinical difference [19, 20]. The study was powered based on the scant literature surrounding the possible proportion of bacteriuria between responders and non‐responders [21]. A sample size of 80 subjects was needed to detect at least a 30% difference in the proportion of patients with ASB between the two groups at a power of 80% with an alpha level of 0.05.

Differences in demographics, comorbidities, and urinary analytes between responders and non‐responders as well as those with ASB and those without ASB were assessed using univariate analysis with *t*‐tests or Wilcoxen tests, and Chi‐Square/Fisher's Exact for continuous and categorical variables, respectively. A *p* value of < 0.05 was considered statistically significant for the demographic variable. A Dunn‐Sidak correction was utilized for comparison of urinary markers.

Decision trees were utilized to identify patterns in the collected subjective and objective data between BTX‐A responders and BTX‐A non‐responders. These decision trees were created using leave one subject out method to split the data and trained the models for each epoch. *A priori* specified features included were age, BMI, diabetes, history of recurrent urinary tract infections, race, menopausal status, concomitant stress urinary incontinence procedure, antibiotic use, units of Botox, pre‐procedure UDI‐6 score, and measured urinary cytokines. ASB was then added to the decision tree analysis to determine its relative contribution.

## 3 Results

### 3.1 Comparison of Those With and Without ASB

We enrolled 78 participants, with an average age of 69.32 ± 12.7 years and an average baseline UDI‐6 of 53 ± 19. Baseline ASB was present in 28 participants (36%). Participants with baseline ASB were older than those without (75.5 ± 7.4 years vs. 64.4 ± 13.2 years, *p* = 0.02, Table 1) but otherwise did not differ in their demographic characteristics, including vaginal estrogen use and history of recurrent UTI. Of the 15 candidate biomarkers, 12 biomarkers were within detectable levels and analyzed. GRO‐α, β‐NGF, and BDNF were present at too low of concentrations to be reliably detected in the multiplex assay. Based on a Mann‐Whitney‐U analysis with Dunn‐Sidak correction, three biomarkers were significantly different between participants with and without ASB: IL‐6, IL‐8, and TNFa (Table 2). On an exploratory subanalysis looking only at those with ASB, there were no differences in these markers between responders and non‐responders in this subgroup.

**Table 1:** Demographic characteristics of participants with and without asymptomatic bacteriuria.

	Asymptomatic bacteriuria (*n* = 28)	No Asymptomatic bacteriuria (*n* = 44)	*p* value
Age, mean (SD), year	75.5 ± 7.4	64.4 ± 13.2	**0.02**
BMI (kg/m^2^) mean (SD)	32.31 ± 7.2	33.53 ± 7.2	0.13
Obese (categorical)	16 (57.1%)	25 (56.82%)	0.99
Race (categorical)			0.63
White	25 (89.3%)	42 (95.5%)	
Black	2 (7.1%)	1 (2.3%)	
Not reported	1 (3.6%)	1 (2.3%)	
Postmenopausal (categorical)	27 (96.4%)	35 (79.5%)	0.08
History of recurrent UTIs (categorical)	5 (17.9%)	2 (4.5%)	0.10
Diabetes (categorical)	8 (28.6%)	8 (18.2%)	0.39
Units of BTX‐A (categorical)			0.99
100	20 (71.4%)	30 (68.2%)	
200	8 (28.6%)	13 (29.5%)	
300	0 (0.0%)	1 (2.3%)	
Baseline Urogenital Distress Inventory SF, mean (SD)	56.1 ± 19.7	51.2 ± 17.2	0.52
Vaginal estrogen use (categorical)	10 (35.7%)	10 (22.8%)	0.28

*Note:* Bold indicates a statistically significant difference. Three participants did not have culture data and thus were excluded from the calculation.

Abbreviations: ASB, asymptomatic bacteriuria; BMI, body mass index; BTX‐A, OnabotulinumtoxinA; SF, Short Form; UTIs, urinary tract infection.

**Table 2:** Mann−Whitney‐U analysis of inflammatory mediator differences in patients with and without ASB.

Cytokine	ASB (*n* = 28) median (IQR)	Non‐ ASB (*n* = 44) median (IQR)	*p* value (MWU)	Significant, Dunn‐Sidak correction for multiple comparisons
bFGF	0.099 (0.72)	0.047 (0.24)	0.121	*p* > 0.05
VEGF	1.960 (1.55)	1.479 (1.58)	0.030	*p* > 0.05
IFNG	0.015 (0.02)	0.010 (0.01)	0.134	*p* > 0.05
IL10	0.004 (0.00)	0.002 (0.00)	0.019	*p* > 0.05
IL12p70	0.004 (0.01)	0.003 (0.01)	0.079	*p* > 0.05
IL13	0.054 (0.07)	0.020 (0.02)	0.005	*p* > 0.05
IL4	0.002 (0.00)	0.001 (0.04)	0.039	*p* > 0.05
IL6	0.035 (0.04)	0.018 (0.00)	0.004	***p*** < **0.05**
IL8	0.205 (0.86)	0.020 (0.04)	0.000	***p*** < **0.05**
TNFA	0.011 (0.01)	0.005 (0.00)	0.003	***p*** < **0.05**
IP10	0.379 (0.71)	0.122 (0.27)	0.004	*p* > 0.05
MCP1	3.718 (3.48)	1.748 (2.29)	0.030	*p* > 0.05

*Note:* Bold indicates a statistically significant difference.

Abbreviations: ASB, asymptomatic bacteriuria; MWU, Mann−Whitney *U* test.

### 3.2 Analysis of BTX‐A Responders

**Figure 1:**
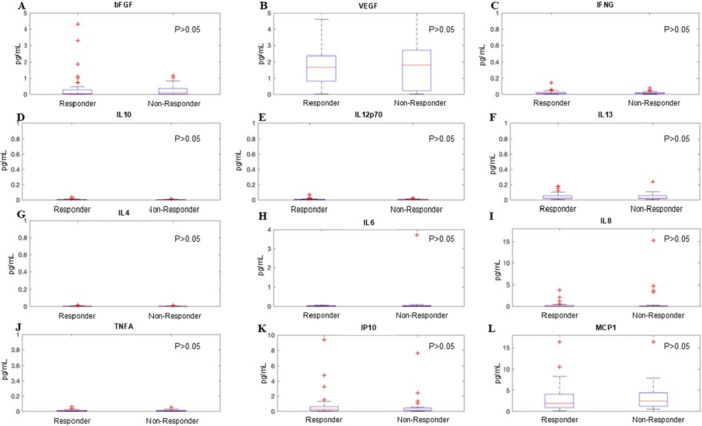
(A−L) Box‐and‐whisker plot of urinary cytokine and chemokine concentrations (pg/mL) in patients that responded to Onabotulinumtoxin‐A for the treatment of idiopathic overactive bladder (responder) compared to those who did not respond to the intervention (non‐responder). Cytokine and chemokine concentrations were normalized to patient urinary creatinine. The red line indicates the median, blue lines indicate the 25th and 75th percentiles, and the whiskers extend to 1.5 times the interquartile range. Outliers are indicated as red crosses.

Of the 75 participants who had post‐injection UDI‐6 data, 45 (60%) were responders, and 30 (40%) were non‐responders (Table 3). Responders were younger than non‐responders (66.0 ± 14.2 years for responders vs. 74.3 ± 7.8 years for non‐responders, *p* < 0.01) but otherwise did not differ in their demographic characteristics. There was no difference in the rate of ASB in responders (37.8%) versus non‐responders (40.7%, *p* = 0.09). There were no significant differences in biomarker concentrations between responders and non‐responders based on Mann−Whitney‐*U* analysis, even when accounting for age (Figure 1). There was no correlation between single analytes measured and pre‐procedure UDI‐6, post‐procedure UDI‐6, or the difference in pre versus post UDI‐6 score.

**Table 3:** Demographic characteristics of responders and non‐responders.

	Responders (*n* = 45)	Non‐responders (*n* = 30)	*p* value
Age, mean (SD), year	66.0 ± 14.2	74.3 ± 7.8	**< 0.01**
BMI (kg/m^2^) mean (SD)	34.2 ± 8.9	31.2 ± 6.7	0.12
Obese (categorical)	29 (64.4%)	14 (46.7%)	0.15
Race (categorical)			0.64
White	41 (91.1%)	29 (96.7%)	
Black	2 (4.4%)	1 (3.3%)	
Postmenopausal (categorical)	37 (82.2%)	28 (9.3%)	0.30
History of recurrent UTIs (categorical)	3 (6.7%)	4 (13.3%)	0.43
ASB (categorical)^a^	17 (37.8%)	11 (40.7%)	0.09
Diabetes (categorical)	11 (24.4%)	6 (20.0%)	0.79
Units of BTX‐A (categorical)			0.90
100	32 (71.1%)	20 (66.7%)	
200	12 (26.7%)	9 (30.0%)	
300	1 (2.2%)	1 (3.3%)	
Baseline Urogenital Distress Inventory SF, mean (SD)	52.04 ± 16.2	53.61 ± 21.1	0.71
Vaginal estrogen use (categorical)	9 (20.0%)	12 (40.0%)	0.07

*Note:* Bold indicates a statistically significant difference.

Abbreviations: ASB, asymptomatic bacteriuria; BMI, body mass index; BTX‐A, OnabotulinumtoxinA; UTIs, urinary tract infection; SF, short form.

a Three participants did not have culture data and thus were excluded from the calculation.

**Figure 2:**
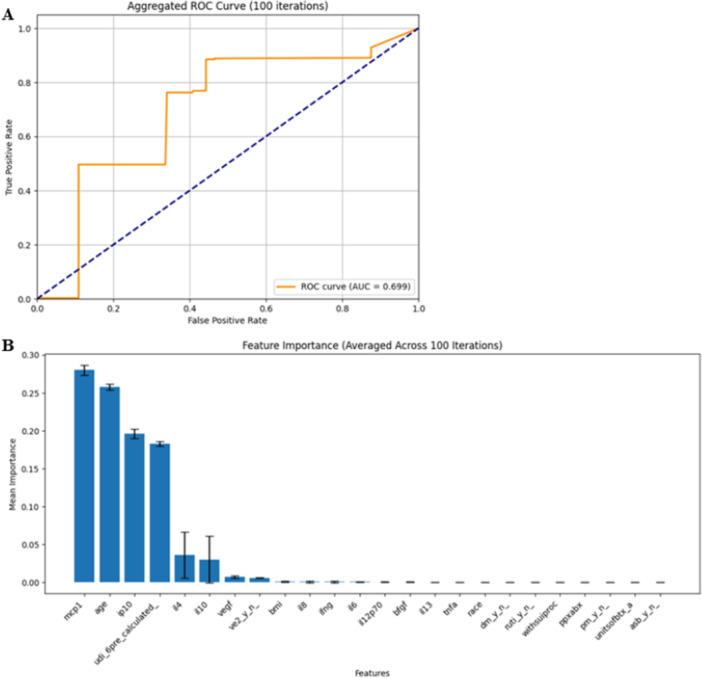
Receiver Operating Characteristic Curve (A) demonstrating sensitivity of 93.3% and specificity of 60% of the final decision tree. Features included in the final decision tree were age, BMI, diabetes, history of recurrent UTIs, race, menopausal status, concomitant SUI procedure, antibiotic use, units of Onabotulinumtoxin‐A, pre‐procedure UDI‐6 score, and measured urinary analytes. Clinical parameters of age and pre‐procedure symptom severity, and urinary cytokines MCP1 and IP10 were the most predictive features. (B) Shows the predictive features of the decision trees, including the presence/absence of ASB.

Decision trees were used to shed qualitative insight into the variables that best predicted response to BTX‐A injection. Including age, BMI, diabetes, history of recurrent urinary tract infections, race, menopausal status, concomitant stress urinary incontinence procedure, antibiotic use, units of BTX‐A, pre‐procedure UDI‐6 score, and measured urinary cytokines produced an accuracy of 80% with a sensitivity of 93.3% and a specificity of 60% (Figure 2A). Clinical parameters of age and pre‐procedure symptom severity and urinary cytokines MCP‐1 and IP‐10 were the most predictive features. Adding ASB to the decision tree analysis did not improve classification nor function as an important predictive feature (Figure 2B), therefore it was not included in the final decision tree. The final decision tree demonstrated that age < 71.5 predicted response, and that for women older than 71.5 years, higher IP10 and lower MCP1 were associated with treatment response. In younger women, lower baseline UDI‐6 and lower IL4 levels were associated with response to treatment (Supporting Information S1: Figure 1).

## 4 Discussion

In this prospective cohort analysis of ASB status and urinary chemokine and cytokine data, we found that (1) neither ASB status nor a narrow analysis of urine inflammatory markers are sufficient in predicting treatment response to BTX‐A; (2) responders were younger than non‐responders, consistent with prior literature; (3) patients with ASB demonstrated an increase in proinflammatory markers compared to those without ASB, although the clinical significance of this is not yet clear and (4) higher IP10 and lower MCP1 were associated with treatment response in older women, while in younger women lower symptom severity and lower IL4 levels predicted response.

Although further studies are needed, our study suggests initial reassurance that ASB should not affect treatment response to BTX‐A. Importantly, this is in line with the literature in neurogenic bladder, where ASB has not been shown to decrease clinical response to BTX‐A [22]. Also consistent with previous literature, age was a significant factor in responder status [5]. Contrary to previous literature [5], there did not appear to be a significant difference in BMI between responder groups; however, this could be due to this study's smaller sample size.

Notably, we defined ASB as presence of bacteriuria on standard culture and so cannot comment on responder status based on other microbes that may have been assessed using PCR‐based technology. Literature supports nuanced differences in the bladder microbiome of individuals with and without ASB [7] and even amongst age groups in individuals with ASB. Older individuals (70+ years of age) and younger individuals (20‐49 years of age) are more likely to have larger amounts of bacteria in their urine than individuals aged 50−69. Moreover, there are significant changes in the bacterial composition in the urine of 70+ year old women, with genera such as *Jonquetella* and *Parvimonas* uniquely present in this population [23]. One recent study suggests that there are, indeed, nuanced microbiome differences both at baseline and over time between responders and non‐responders to BTX‐A, and this should be further studied [24]. It is also possible that BTX‐A injection in patients with ASB or an altered microbiome could increase the risk of UTI post‐injection and confound subjective symptom relief [25]; however, a secondary analysis of the ROSETTA trial found no difference in treatment response based on the presence or absence of a UTI after BTX‐A injection [26].

We also found numerous differences in cytokine and chemokine concentration in participants with and without ASB, primarily higher concentrations of pro‐inflammatory cytokines. These results can inform work seeking a biomarker to distinguish true UTI and those with ASB, which has been difficult, perhaps due to a proinflammatory state in both conditions [27].

The lack of a single biomarker to predict treatment response seen here is consistent with an evolving literature base that struggles to find a set of quantitative urodynamic biomarkers that differentiates subjects who respond to BTX‐A [11, 18]. The most investigated biomarkers of OAB are urinary cytokines and chemokines, including nerve growth factor (NGF) and c‐reactive protein (CRP), amongst others. Attempts to identify reliable biomarkers of OAB response have so far been limited by the variability of OAB symptoms in subjects and inadequate predictive value [9, 28]. One study implicated higher NGF in poor BTX‐A treatment response, but failure to control for possible ASB amongst participants may have confounded the strength of their results [22]. In addition, our study looked only at baseline markers at one time point. Cytokines and chemokines are known to be dynamic in response to bacteria, stress, and other triggers over time. Looking at multiple time points or using a systems biology approach to allow better integration of multiple complex markers may be fruitful future directions.

Decision trees have the potential to guide clinicians in identifying BTX‐A responders from non‐responders based on urinary biomarkers. This study highlights the difference in younger vs older women with OAB, as different features predicted response for each group. In older women, treatment response demonstrated lower MCP‐1, a monocyte attractant, and low IP10, which participates in T cell activation and migration. This may indicate that a higher innate immune response at the time of BTX‐A injection is detrimental to therapy. In younger women, baseline symptom severity as measured on UDI‐6 was critical. Interestingly, higher IL‐4, traditionally associated with immunomodulation, was associated with non‐responders, which deserves further investigation.

The strengths of this study include the use of both ASB status and urinary cytokine data in evaluating differences in BTX‐A treatment response. Additionally, the use of decision tree analysis in this setting highlights opportunities for further investigation of a subset of inflammatory mediators and clinical factors that appear to be most important in differentiating responders from non‐responders. Limitations of this study include the relatively small dataset, lack of plasma biomarkers to corroborate urinary biomarker trends, and lack of longitudinal data. Method of sample collection (catheter vs. voided) may influence inflammatory cytokines measured, and this should be considered in comparing these findings to other studies. Finally, we did not collect data on whether patients were BTX‐A‐naive or the time since the last injections. It is known that BTX‐A injections can result in at least transient changes in the microbiome and inflammatory cytokines, however the duration of these changes is not well understood. This will be further explored in future studies.

It should be noted that due to a greater loss‐to‐follow‐up rate than anticipated, we did not meet our planned total sample size of 80. However, the observed difference between ASB rates in responders versus non‐responders was only 2.9%. It would require nearly 12 000 total patients to demonstrate this as a statistically significant difference, and such as small difference is unlikely to represent a clinically significant difference. Confirmatory analyses on a larger sample size would be important to more fully comprehend the impact of both ASB and biomarkers on treatment response to BTX‐A.

## 5 Conclusion

These data suggest that there is no significant difference in treatment response to BTX‐A injection for the treatment of refractory OAB based on the presence or absence of baseline ASB on standard culture, which can reassure clinicians that such testing is unnecessary. Older women were less likely to respond to BTX‐A treatment for OAB. Decision trees indicate low MCP1 and high IP10 as associated with treatment response in older women, highlighting the roles of innate and adaptive immunity and the importance of biomarker combinations in predicting treatment response.

## Author Contributions

**Marina Guirguis Hanna:** conceptualization, investigation, and manuscript writing. **Ashti M. Shah:** investigation, formal analysis, manuscript writing, and visualization. **Wuqi Li:** methodology, validation, formal analysis, visualization, and manuscript writing. **Ervin Sejdic:** methodology, validation, formal analysis, and manuscript editing. **Megan Bradley:** conceptualization, investigation, manuscript editing. **Stephanie W. Zuo:** investigation, manuscript editing. **Amanda M. Artsen:** conceptualization, methodology, validation, formal analysis, supervision, and manuscript editing.

## Disclosure

The authors have nothing to report.

## Ethics Statement

This study was approved by the University of Pittsburgh Institutional Review Board (#21010132). All participants provided informed consent.

## Conflicts of Interest

The authors declare no conflicts of interest.

## Supporting information


**Supplemental Figure 1:** Final decision tree plotted from a model trained by all the data. **Supplemental Table 1:** Limits of Quantification.

## Data Availability

Data will be made available on request.
