# Supplementary material for: Urinary Cytokines in Predicting Intradetrusor Onabotulinumtoxin‐A Response
Source: Neurourol Urodyn. 2026 Mar 15;45(4):845–52. doi: 10.1002/nau.70261 (PMC13054632; doi:10.1002/nau.70261)
Supplement: Supplementary file 1 — Supplemental Figure 1: Final decision tree plotted from a model trained by all the data. Supplemental Table 1: Limits of Quantification. [file NAU-45-845-s001.docx]

**Supplemental Table 1. Limits of Quantification.**

| Analyte | Lower Limit of Quantitation | Upper Limit of Quantitation | MSD Panel |
| --- | --- | --- | --- |
| IFNγ | 1.76 | 938 | Proinflammatory |
| IL-10 | 0.298 | 233 | Proinflammatory |
| IL-12p70 | 1.22 | 315 | Proinflammatory |
| IL-13 | 4.21 | 353 | Proinflammatory |
| IL-4 | 0.218 | 158 | Proinflammatory |
| IL-6 | 0.633 | 488 | Proinflammatory |
| IL-8 | 0.591 | 375 | Proinflammatory |
| TNFα | 0.690 | 248 | Proinflammatory |
| VEGF | 7.70 | 562 | Proinflammatory |
| bFGF | 2.60 | 1780 | Angiogenesis |
| IP-10 | 1.37 | 500 | Chemokine |
| MCP-1 | 1.09 | 375 | Chemokine |
| GRO-α | 0.29 | 12276 | Custom U-Plex |
| β-NGF | 0.30 | 4759 | Custom U-Plex |
| BDNF | 3.52 | 17338 | Custom U-Plex |

Values are given in pg/mL. Custom U-Plex limits were determined experimentally, all other analyte ranges are provided by the manufacturer during validation.

**
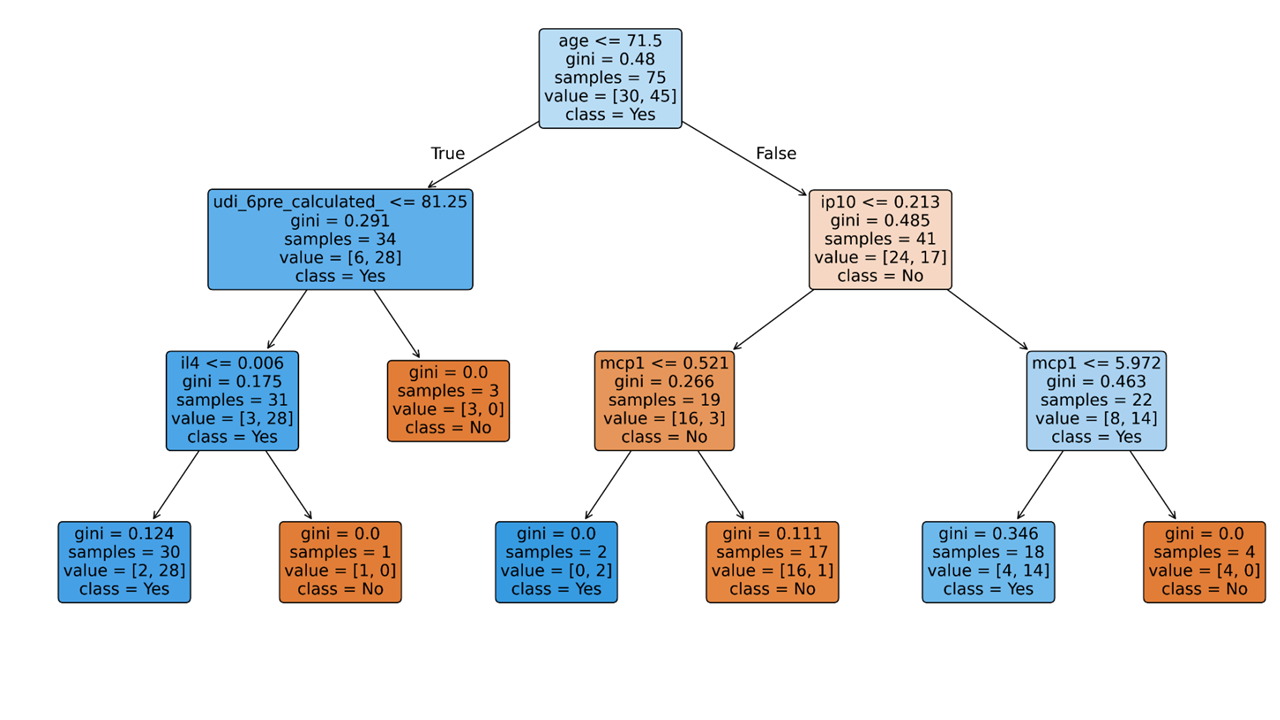
**

**Supplemental Figure 1**. Final decision tree plotted from a model trained by all the data. For each node, “gini” denotes the Gini Impurity Index, “value” indicates the count of cases for each class (no/yes), “samples” specifies the total number of cases in the node, and “class” identifies the most probable class for the node." Importance of MCP1 and IP10 are demonstrated in older women while clinical symptom severity as measured by baseline UDI-6 and IL4 are demonstrated in younger women.
